# Albuminuria as a predictor of mortality in type II diabetic patients after living-donor liver transplantation

**DOI:** 10.1080/07853890.2022.2124446

**Published:** 2022-09-26

**Authors:** Ahmed Abdallah Salman, Mohamed Abdalla Salman, Mostafa Said, Hesham Elkassar, Mohammad El Sherbiny, Ahmed Youssef, Mohammed Elbaz, Ahmed M. Elmeligui, Mohamed Badr Hassan, Mahmoud Gouda Omar, Hussien Samir, Mohamed Abdelkader Morad, Hossam El-Din Shaaban, Mohamed Youssef, Ahmed Moustafa, Mohamed Sabry Tourky, Ahmed Elewa, Sadaf Khalid, Khaled Monazea, Mohamed Shawkat

**Affiliations:** a Internal Medicine Department, Faculty of Medicine, Cairo University, Cairo, Egypt; b General Surgery Department, Faculty of Medicine, Cairo University, Cairo, Egypt; c Gastroenterology Department, National Hepatology and Tropical Medicine Research Institute, Cairo, Egypt; d Department of Endemic Medicine and Hepatology, Faculty of Medicine, Cairo University, Cairo, Egypt; e Department of Surgery, Great Western Hospitals NHS Foundation Trust, Swindon, UK; f General Surgery Department, National Hepatology and Tropical Medicine Research Institute, Cairo, Egypt; g General Surgery Department, Royal Free Hospital, London, UK; h General Surgery Department, Assiut Faculty of Medicine for Boys, Al-Azhar University, Cairo, Egypt; i Internal Medicine Department, Faculty of Medicine, Minia University, Minia, Egypt

**Keywords:** Liver transplant, albuminuria, diabetes

## Abstract

**Purpose:**

Diabetes mellitus (DM) increases the risk of morbidity and mortality after liver resection. Albuminuria is associated with a higher risk for all-cause and cardiovascular mortality. This study evaluated albuminuria as a predictor of the outcome of living donor liver transplantation (LDLT) in patients with pre-existing DM.

**Methods:**

This retrospective study involved 103 type II diabetic patients with end-stage liver disease who received LDLT. Preoperative spot urine albumin: creatinine ratio was used to determine the degree of albuminuria. The primary outcome measure was the impact of urinary albumin excretion on the 3-year mortality rate after LDLT in this diabetic cohort.

**Results:**

Hepatitis C virus infection was the main cause of cirrhosis. Albuminuria was detected in 41 patients (39.8%); 15 had macroalbuminuria, while 26 had microalbuminuria. Patients with microalbuminuria were significantly older than those with macroalbuminuria and normal albumin in urine. After 3 years, twenty-four patients (23.3%) died within 3 years after LT. Myocardial infarction was the leading cause of death (25%). Albuminuria was an independent factor affecting 3-year mortality with an odds ratio of 5.17 (95% CI: 1.86–14.35).

**Conclusion:**

Preoperative albuminuria is an independent factor affecting mortality within 3 years after LDLT in type II diabetic patients. Myocardial infarction was the leading cause of death in 25% of cases, followed by hepatocellular carcinoma recurrence, sepsis, and graft failure.

**KEY MESSAGES**
- Diabetes mellitus (DM) increases the risk of morbidity and mortality after liver resection.
- Albuminuria is associated with a higher risk for all-cause and cardiovascular mortality.
- Preoperative albuminuria is a significant predictor of mortality within 3 years after LDLT in diabetic patients.

## Introduction

In Egypt, 13 centres perform living donor liver transplantation (LDLT). By June 2014, 2,406 procedures were done. The main indication was cirrhosis post-hepatitis-C viral infection [1]. Deceased donor liver transplant has not yet been authorised due to religious and cultural factors [2]. Patient survival after liver transplantation (LT) has markedly improved thanks to the modifications in surgical techniques and postoperative care [3]. However, the impact of associated morbidities on the outcome of LT varies widely [4].

Type II diabetes mellitus (T2DM) is frequently detected in patients with severe liver diseases, including cirrhosis and hepatocellular carcinoma (HCC) [5,6] that may require LT in the terminal stage. It was found that T2DM increases the risk of morbidity and mortality after liver resection for HCC and liver metastasis [7]. It was reported that at the time of LT, 33–50% of the patients had renal dysfunction, and 6.3% were receiving dialysis [8,9]. Albuminuria is widely recognised as a marker for kidney disease in healthy subjects and patients with diabetes, hypertension, and obesity [10]. Higher albuminuria degrees are associated with a higher risk for all-cause and cardiovascular mortality and chronic kidney disease evolution [11].

This study aimed to evaluate albuminuria as an early marker for renal dysfunction in patients with pre-existing T2DM for predicting the outcome of LDLT.

## Patients and methods

This retrospective analysis was performed from May 2005 to June 2012. All type II diabetic patients with end-stage liver disease who received LDLT were enrolled. Patients <18 years of age, having a previous liver transplant, with non-diabetic causes of albuminuria, and those with elevated serum creatinine above 1.2 and 1.4 mg for females and males, respectively, and/or end-stage renal disease were excluded. The study protocol adhered to the ethical guidelines of the 1975 Declaration of Helsinki. The local ethical committee of Cairo University Hospitals approved the protocol and patients’ consent was waived due to the retrospective nature of the study.

All patients’ medical records were reviewed to extract the following data: demographic information, aetiology of primary liver disease, clinical and laboratory parameters, graft–recipient weight ratio, donor age and degree of steatosis, duration of hospital and ICU stay, and outcome within 3 years.

The severity of liver disease was assessed by Child-Pugh points [12] and model for end-stage liver disease (MELD) score [13]. T2DM was diagnosed according to the American diabetes association criteria based on fasting plasma glucose (FPG) level. Clinical diabetes mellitus (DM) was defined as FPG 126 mg/dL or HbA1c 6.5%. This was confirmed by repeated blood sampling unless the patient has clinical symptoms or a glucose level of 200 mg/dL, previous history of DM, and/or consumption of anti-diabetes medications [14].

Determination of micro- and macro-albuminuria was based on preoperative spot urine albumin creatinine ratio (uACR) measurements. Data from early morning urine samples were used. Normoalbuminuria was defined as uACR ≤2.5 mg/mmol for males and ≤3.5 mg/mmol for females. Microalbuminuria was defined as uACR >2.5–30 mg/mmol for males and >3.5–30 mg/mmol for females. Macroalbuminuria was defined as uACR >30 mg/mmol [15]. eGFR was computed using the modified Modification of Diet in Renal Disease formula [16].

The primary outcome measure was the impact of urinary albumin excretion on the 3-year mortality rate after LDLT in this type II diabetic cohort.

### Statistical analysis

Statistical analysis was done using IBM© SPSS© Statistics version 23 (IBM© Corp., Armonk, NY, USA). Numerical data were expressed as mean and standard deviation or median and range as appropriate. Qualitative data were expressed as frequency and percentage. Chi-square test was used to examine the relation between qualitative variables. For quantitative data, comparison between two groups was made using independent sample *t*-test or Mann–Whitney test. Comparison between three groups was made using ANOVA test, or Kruskal–Wallis test followed by the appropriate *post hoc* test. Odds ratio (OR) with its 95% confidence interval (CI) were used for risk estimation. A *p*-value <.05 was considered significant.

## Results

A total of 103 patients were enrolled in the study. According to the level of uACR, albuminuria was detected in 41 patients (39.8%); 15 had macroalbuminuria, while 26 had microalbuminuria. Table 1 presents a comparison between the three groups; macroalbuminuria, microalbuminuria, and no albuminuria. Patients with macroalbuminuria were significantly older than those without albuminuria (*p* = .014) and had comparable age with the microalbuminuria group (*p* = .234). Microalbuminuria and no albuminuria groups were comparable in recipient age (*p* = .443). Otherwise, there was no significant difference between the three groups in all demographic, clinical, and laboratory characteristics of the patients (Table 1).

**Table 1.** Comparison between patients with macroalbuminuria, microalbuminuria, and no albuminuria regarding demographic, clinical, and laboratory characteristics.

	Macroalbuminuria	Microalbuminuria	No albuminuria	*p*-Value
	*n* = 15	*n* = 26	*n* = 62	
Recipient age (years)	52.4 ± 6.7	49.4 ± 5.6	47.8 ± 4.9	**.012**
Donor age (years)	26.3 ± 3.7	29.6 ± 5.9	28.5 ± 5	.142
Sex				
Male	14 (93.3%)	18 (69.2%)	52 (83.9%)	.154
Female	1 (6.7%)	8 (30.8%)	10 (16.1%)	
Smoking	5 (33.3%)	13 (50.0%)	24 (38.7%)	.504
Body mass index (kg/m^2^)	26.7 ± 2.9	26.3 ± 3.2	27.8 ± 2.6	.060
Duration of DM (years)	8 (2–18)	8 (2–21)	11 (2–24)	.124
Insulin therapy	7 (46.7%)	9 (34.6%)	30 (48.4%)	.488
Oral antidiabetic	13 (86.7%)	22 (84.6%)	47 (75.8%)	.493
Hypertension	6 (40.0%)	4 (15.4%)	15 (24.2%)	.208
Dyslipidaemia	4 (26.7%)	6 (23.1%)	14 (22.6%)	.945
Peripheral vascular disease	1 (6.7%)	3 (11.5%)	2 (3.2%)	.266
Cardiomyopathy	1 (6.7%)	2 (7.7%)	1 (1.6%)	.217
Child-Turcotte-Pugh class				
B	3 (20.0%)	8 (30.8%)	21 (33.9%)	.581
C	12 (80.0%)	18 (69.2%)	41 (66.1%)	
Cause of cirrhosis				
Post-HCV	11 (73.3%)	20 (76.9%)	42 (67.7%)	.496
Post-HBV	3 (20.0%)	2 (7.7%)	14 (22.6%)	
Others	1 (6.7%)	4 (15.4%)	6 (9.7%)	
Hepatocellular carcinoma	3 (20.0%)	4 (15.4%)	13 (21.0%)	.832
MELD score	17.0 ± 3.4	18.1 ± 3.9	17.5 ± 4.3	.685
Graft-to-recipient weight ratio (%)	1.14 ± 0.2	1.17 ± 0.13	1.22 ± 0.17	.144
Donor fatty changes by liver biopsy (%)	8.7 ± 2.7	8.7 ± 2.0	9.6 ± 2.3	.195
uACR (mg/mmol)	39.0 (34.0–54.0)	17.0 (6.0–24.0)	1.9 (0.4–3.0)	<.001
eGFR (mL/min/1.73 m^2^)	94.3 ± 11.4	93.4 ± 8.7	95.7 ± 8.0	.505
FBS (mg/dL)	145.7 ± 43.5	139.7 ± 32.6	155.3 ± 44.1	.254
HbA1c (%)	7.6 ± 0.8	7.7 ± 0.9	7.7 ± 0.9	.832
ALT (U/L)	48.3 ± 18.8	42.6 ± 14.4	44.6 ± 11.9	.450
AST (U/L)	65.9 ± 24.5	57.8 ± 17.4	58.1 ± 12.5	.214
Serum creatinine (mg/dL)	1.1 ± 0.2	1.0 ± 0.2	1.1 ± 0.2	.135

Data are expressed as mean ± SD, number (%), or median (range).

Bold value signify, *p* < 0.01.

MELD: model for end-stage liver disease; uACR: urine albumin-to-creatinine ratio; eGFR: estimated glomerular filtration rate (mL/min/1.73 m^2^); FBS: fasting blood sugar; HbA1c: glycated haemoglobin (%); ALT: alanine aminotransferase; AST: aspartate aminotransferase.

**Figure 1.**
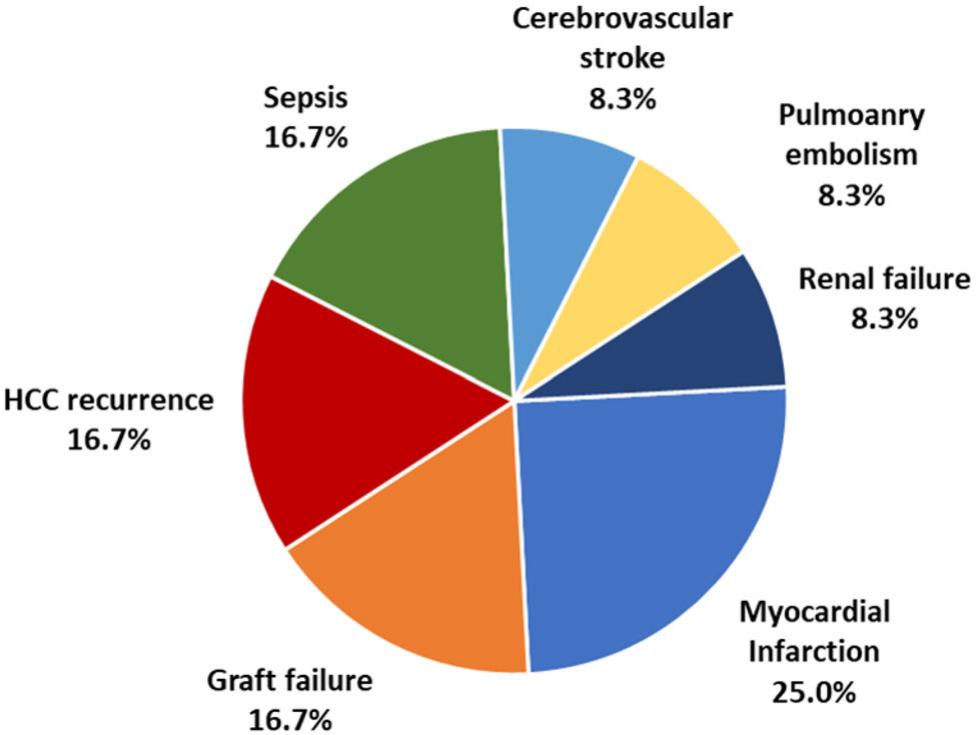
Cause of death within 3 years in 24 patients.

At 3 years, 24 patients (23.3%) died, and myocardial infarction was the leading cause of death (25%) (Figure 1). Albuminuria, whether micro- or macroalbuminuria, was associated with significantly higher 3-year mortality (Table 2). Three-year mortality was also higher in patients with cardiomyopathy (*p* = .039). On multivariate analysis, albuminuria was the only independent factor affecting 3-year mortality (Table 3).

**Table 2.** Factors associated with 3-year mortality in the studied group.

	Non-survivors *n* = 24	Survivors *n* = 79	*p*-Value
Albuminuria			
Macro	6 (25.0%)	9 (11.4%)	**.002**
Micro	11 (45.8%)	15 (19.0%)	
Normal	7 (29.2%)	55 (69.6%)	
Recipient age (years)	50.1 ± 6.7	48.5 ± 5.1	.231
Donor age (years)	30 ± 5.8	28 ± 4.9	.093
Sex			
Male	18 (75.0%)	66 (83.5%)	.374
Female	6 (25.0%)	13 (16.5%)	
Smoking	10 (41.7%)	32 (40.5%)	.919
Body mass index (kg/m^2^)	27.1 ± 3.4	27.4 ± 2.7	.690
Duration of DM (years)	9 ± 5.3	10.1 ± 4.4	.314
Insulin therapy	11 (45.8%)	35 (44.3%)	.895
Oral antidiabetic	18 (75.0%)	64 (81.0%)	.567
Hypertension	4 (16.7%)	21 (26.6%)	.321
Dyslipidaemia	5 (20.8%)	19 (24.1%)	.744
Peripheral vascular disease	0 (0.0%)	6 (7.6%)	.332
Cardiomyopathy	3 (12.5%)	1 (1.3%)	.039
Child-Turcotte-Pugh class			
B	8 (33.3%)	24 (30.4%)	.784
C	16 (66.7%)	55 (69.6%)	
Cause of cirrhosis			
Post-HCV	17 (70.8%)	56 (70.9%)	.395
Post-HBV	6 (25.0%)	13 (16.5%)	
Others	1 (4.2%)	10 (12.7%)	
Hepatocellular carcinoma	5 (20.8%)	15 (19.0%)	1.000
MELD score	18 ± 4.9	17.5 ± 3.8	.585
Graft-to-recipient weight ratio (%)	1.2 ± 0.1	1.2 ± 0.2	.234
Donor fatty changes by liver biopsy (%)	9 ± 2.4	9.3 ± 2.2	.571
eGFR (mL/min/1.73 m^2^)	94.4 ± 10.6	95.1 ± 8.1	.789
Fasting blood sugar (mg/dL)	140 ± 38.2	153 ± 42.3	.181
Glycated haemoglobin (%)	7.8 ± 0.7	7.7 ± 0.9	.697
Alanine aminotransferase (U/L)	47.2 ± 15.2	43.8 ± 13.2	.294
Aspartate aminotransferase (U/L)	64.1 ± 19	57.7 ± 14.9	.086
Serum creatinine (mg/dL)	1.1 ± 0.2	1.1 ± 0.2	.347

Data are expressed as mean ± SD, or number (%). Bold value signify, *p* < 0.01.

MELD: model for end-stage liver disease; eGFR: estimated glomerular filtration rate.

**Table 3.** Multivariate logistic regression model for factors affecting 3-year mortality.

	*B*	*p*-Value	Odds ratio	95% CI for odds ratio
Albuminuria 3-categories model				
Albuminuria		.007		
Macroalbuminuria vs. Normal	1.591	.019	4.91	1.30–18.51
Microalbuminuria vs. Normal	1.672	.004	5.32	1.72–16.47
Cardiomyopathy	2.104	.094	8.20	0.70–96.02
Albuminuria 2-categories model				
Albuminuria	1.642	.002	5.17	1.86–14.35
Cardiomyopathy	2.105	.094	8.21	0.70–96.09

*B*: regression coefficient.

Table 4 compares patients with any degree of albuminuria and non-albuminuric patients. Patients with albuminuria were significantly older (*p* = .021) and had lower BMI (*p* = .019) and shorter duration of DM (*p* = .028). Seventeen patients (70.8%) of those who died within 3 years had albuminuria. Albuminuria was an independent factor affecting 3-year mortality with an OR of 5.17 (95% CI: 1.86–14.35).

**Table 4.** Comparison between patients with albuminuria and those with no albuminuria regarding demographic, clinical, and laboratory characteristics.

	Albuminuria *n* = 41	No Albuminuria *n* = 62	*p*-Value
Recipient age (years)	50.5 ± 6.1	47.8 ± 4.9	.021
Donor age (years)	28.4 ± 5.4	28.5 ± 5	.898
Sex			.456
Male	32 (78.0%)	52 (83.9%)	
Female	9 (22.0%)	10 (16.1%)	
Smoking	18 (43.9%)	24 (38.7%)	.600
Body mass index (kg/m^2^)	26.5 ± 3.1	27.8 ± 2.6	.019
Duration of DM (years)	8 (2–21)	11 (2–24)	.028
Insulin therapy	16 (39.0%)	30 (48.4%)	.349
Oral antidiabetic	35 (85.4%)	47 (75.8%)	.238
Hypertension	10 (24.4%)	15 (24.2%)	.982
Dyslipidaemia	10 (24.4%)	14 (22.6%)	.832
Peripheral vascular disease	4 (9.8%)	2 (3.2%)	.213
Cardiomyopathy	3 (7.3%)	1 (1.6%)	.299
Child-Turcotte-Pugh class			.450
B	11 (26.8%)	21 (33.9%)	
C	30 (73.2%)	41 (66.1%)	
Cause of cirrhosis			.406
Post-HCV	31 (75.6%)	42 (67.7%)	
Post-HBV	5 (12.2%)	14 (22.6%)	
Others	5 (12.2%)	6 (9.7%)	
Hepatocellular carcinoma	7 (17.1%)	13 (21.0%)	.625
MELD score	17.7 ± 3.7	17.5 ± 4.3	.831
Graft-to-recipient weight ratio (%)	1.2 ± 0.2	1.22 ± 0.17	.053
Donor fatty changes by liver biopsy (%)	8.7 ± 2.2	9.6 ± 2.3	.070
uACR (mg/mmol)	23.0 (6.0–54.0)	1.9 (0.4–3.0)	<.001
eGFR (mL/min/1.73 m^2^)	93.7 ± 9.6	95.7 ± 8.0	.259
FBS (mg/dL)	141.9 ± 36.5	155.3 ± 44.1	.110
HbA1c (%)	7.7 ± 0.8	7.7 ± 0.9	.744
ALT (U/L)	44.7 ± 16.2	44.6 ± 11.9	.971
AST (U/L)	60.8 ± 20.3	58.1 ± 12.5	.454
Serum creatinine (mg/dL)	1.1 ± 0.2	1.1 ± 0.2	.115

Data are expressed as mean ± SD, number (%), or median (range).

MELD: model for end-stage liver disease; uACR: urine albumin-to-creatinine ratio; eGFR: estimated glomerular filtration rate (mL/min/1.73 m^2^); FBS: fasting blood sugar; HbA1c: glycated haemoglobin (%); ALT: alanine aminotransferase; AST: aspartate aminotransferase.

Table 5 shows causes of death in patients with macroalbuminuria, microalbuminuria, and no albuminuria.

**Table 5.** Causes of death in patients with macroalbuminuria, microalbuminuria, and no albuminuria.

	Macroalbuminuria	Microalbuminuria	No Albuminuria
	*n* = 6	*n* = 11	*n* = 7
Myocardial infarction	0 (0.0%)	3 (27.3%)	3 (42.9%)
Sepsis	1 (16.7%)	3 (27.3%)	0 (0.0%)
HCC recurrence	1 (16.7%)	0 (0.0%)	3 (42.9%)
Graft failure	1 (16.7%)	2 (18.2%)	1 (14.3%)
Pulmonary embolism	1 (16.7%)	1 (9.1%)	0 (0.0%)
Cerebrovascular stroke	2 (33.3%)	0 (0.0%)	0 (0.0%)
Renal failure	0 (0.0%)	2 (18.2%)	0 (0.0%)

## Discussion

This study found that preoperative detection of albuminuria was significantly associated with mortality within 3 years after LDLT in type II diabetic patients. Myocardial infarction was the leading cause of death in 25% of cases, followed by HCC recurrence, sepsis, and graft failure.

In Egypt, T2DM is a rapidly growing health problem affecting about 15.6% of adults. It is the primary cause of end-stage renal disease in Egypt [17]. In this retrospective study, we investigated the predictive value of urinary albumin excretion in diabetic patients with liver cirrhosis subjected to LDLT for mortality within 3 years after surgery. In 70% of the current series, cirrhosis was due to viral hepatitis infection, whether hepatitis C virus (HCV) or HBV. Liver cirrhosis is commonly associated with DM. It is estimated that DM is present in 12.3–57.0% of patients with cirrhosis [18]. Also, patients with HCV were found to be more prone to develop T2DM [19]. The coexistence of DM and chronic liver diseases are associated with a higher risk of hepatic decompensation, HCC, and mortality [20].

In patients with end-stage liver disease, DM positively correlates with disease severity [21,22]. Many studies reported lower survival in decompensated liver disease in patients with DM [23]. The presence of diabetes was associated with about a 50% greater risk of mortality in patients with CLD [24]. Pre-existing DM was shown as an independent factor for developing ascites, bacterial infections, and renal dysfunction in patients with post-HCV cirrhosis [25].

In terms of LT, pre-transplant DM is an important predictor of post-transplant DM and metabolic syndrome [26,27]. The presence of post-transplant metabolic syndrome increases the incidence of cardiovascular events [28].

The effect of pre-existing DM on survival after LT has been previously investigated with controversial results. Some studies did not find an association between DM before LT and survival after LT [29–31]. On the other hand, John and Thuluvath reported significant post-LT morbidity in patients with pre-existing DM [32]. The Scientific Registry of Transplant Recipients (SRTR) database demonstrated that pre-existing DM increased post‐transplant mortality risk by 20% [33]. Reduced survival was mainly found in patients requiring insulin or having underlying chronic hepatitis C infection [34,35].

Albuminuria was demonstrated as a predictor of mortality in diabetic patients of Asian and Caucasian populations [36,37]. It was found to be associated with in-hospital deaths of diabetic patients with foot complications [38]. It was also associated with a higher risk of all-cause and cardiovascular mortality than those without albuminuria after long-term follow-up [39]. Another meta-analysis of 148,350 cases confirmed these findings in diabetic patients with microalbuminuria and macroalbuminuria [40].

Moreover, even with low concentrations, albuminuria has been recognized as a critical cardio-renal risk marker [41]. Albumin excretion in urine indicates glomerular and tubular damage resulting in functional renal impairment [42,43]. On the other hand, the link between renal and cardiovascular damage is explained by the fact that endothelial damage indicated by albuminuria leads to increased systemic vascular permeability. Endothelial damage causes increased cardiovascular risk [44]. Diabetic patients with albuminuria are more prone to developing myocardial infarction and stroke compared with the general population [45]. Moreover, a previous study had demonstrated that preoperative proteinuria may anticipate the occurrence of renal injury and that can affect mortality in those undergoing cardiac surgery [46]. In addition, it was reported that proteinuria was linked to postoperative acute kidney injury and 30-day unplanned readmission independent of preoperative eGFR [47].

The current work found that preoperative albuminuria was significantly associated with mortality post LDLT. Previous studies have tried to investigate the effect of albuminuria on LT. Pan and colleagues found that the presence of proteinuria ahead of LT is a negative predictor for in-hospital survival. In addition, they reported that the existence of proteinuria after LT was an early prognostic factor of the short-term outcome. Based on these findings, repeated surveying of proteinuria in the preoperative and postoperative periods was advocated [48].

Moreover, Amygdalos and his colleagues studied 390 LT recipients and demonstrated that preoperative proteinuria is a reliable tool to predict mortality in LT recipients [49].

The present study has some limitations. The retrospective nature of the study was the main limitation of the present study. However, the relatively large number of the study sample and the reasonably acceptable follow-up period are points of strength that can support the findings. Another limitation was the heterogenous causes of liver disease that indicated LT. However, to our knowledge, only a few studies addressed the impact of urinary albumin excretion in diabetic patients, especially after LDLT. Therefore, this article adds momentum to the literature regarding this unique topic. Furthermore, it is sensible to say that this work may point to nongraft potential contributing factors that may affect prognosis after LT and may provide novel horizons in terms of this aspect. This can make it an attractive goal for therapeutic approaches to focus on detecting albuminuria to improve the outcome after LT. Most importantly, given the statistical results, the study underlines that urinary albumin excretion has a substantial impact on mortality after LT.

In summary, pre-existing albuminuria in diabetic patients subjected to LDLT is a significant predictor of mortality within 3 years. In this series, myocardial infarction was the leading cause of death in 25% of cases, followed by HCC recurrence, sepsis, and graft failure. Although albuminuria is an early marker of renal disease, it is—more notably—a significant predictor of all-cause and cardiovascular mortality and LT.

## Data Availability

The data that support the findings of this study are available from the corresponding author, Salman Ahmed, upon reasonable request.
